# Identification of inflammatory clusters in long-COVID through analysis of plasma biomarker levels

**DOI:** 10.3389/fimmu.2024.1385858

**Published:** 2024-04-30

**Authors:** Shaurya Dhingra, Jia Fu, Gavin Cloherty, Patrick Mallon, Haimanot Wasse, James Moy, Alan Landay, Grace Kenny

**Affiliations:** ^1^ College of Medicine, University of Illinois at Chicago, Chicago, IL, United States; ^2^ Department of Medicine, Rush University Medical Center, Chicago, IL, United States; ^3^ Abbott Laboratories, Abbott Park, IL, United States; ^4^ Centre for Experimental Pathogen Host Research, University College Dublin, Dublin, Ireland

**Keywords:** long COVID, post-acute sequelae of SARS-CoV-2, immune dysregulation, inflammatory clusters, symptom clusters

## Abstract

Mechanisms underlying long COVID remain poorly understood. Patterns of immunological responses in individuals with long COVID may provide insight into clinical phenotypes. Here we aimed to identify these immunological patterns and study the inflammatory processes ongoing in individuals with long COVID. We applied an unsupervised hierarchical clustering approach to analyze plasma levels of 42 biomarkers measured in individuals with long COVID. Logistic regression models were used to explore associations between biomarker clusters, clinical variables, and symptom phenotypes. In 101 individuals, we identified three inflammatory clusters: a limited immune activation cluster, an innate immune activation cluster, and a systemic immune activation cluster. Membership in these inflammatory clusters did not correlate with individual symptoms or symptom phenotypes, but was associated with clinical variables including age, BMI, and vaccination status. Differences in serologic responses between clusters were also observed. Our results indicate that clinical variables of individuals with long COVID are associated with their inflammatory profiles and can provide insight into the ongoing immune responses.

## 1 Introduction

A large proportion of individuals infected by severe acute respiratory syndrome coronavirus 2 (SARS-CoV-2) have one or more symptoms persisting for several months (1, 2). These symptoms can be wide-ranging and most commonly include fatigue, pulmonary abnormalities, neurological impairments, and reduced mobility (3). These post-acute sequalae of SARS-CoV-2, also known as “long COVID”, have risk factors including female sex, older age, minority racial group, and higher body mass index (BMI) (4, 5). Long COVID is less likely to develop in vaccinated individuals (6, 7), and vaccination may improve long COVID symptoms (8–11). Presently, mechanisms underlying long COVID remain poorly understood. Hypothesized mechanisms include immune dysregulation, an inadequate anti-SARS-CoV-2 serologic response, the persistence of a viral reservoir, and the development of autoantibodies (1).

Immune dysregulation has been studied extensively in acute cases of severe COVID-19, with a range of inflammatory markers associated with increased risk of progression to more severe disease (12–14). In the context of long COVID, a variety of immune changes in have been demonstrated including higher levels of IL-4 and IL-6 producing CD4+ T cells, and higher serum levels of IL-1β, IL-6, TNFα, and IP-10 suggesting chronic immune activation (15–17), but no consistent pattern of immune abnormalities has been identified. An increasing body of research has focused on defining clinical phenotypes of long COVID given the marked heterogeneity in clinical presentation, but whether different inflammatory profiles underlie these clinical phenotypes is less well studied (18, 19).

To better understand the unique immune patterns in individuals with long COVID, we used an unsupervised hierarchical clustering approach to derive inflammatory clusters in a cohort of individuals with long COVID and explored the association of these profiles with symptoms and clinical variables.

## 2 Methods

### 2.1 Participants and sample collection

This study was approved by the Rush University Institutional Review Board, Office of Research Affairs# 20032309. Patients were enrolled for this study at Rush Post COVID clinic, where samples were collected. All enrolled participants were included in this study. Samples were then centrifuged to isolate plasma which was stored at -80 degrees C. Participant electronic medical records (EMRs) were reviewed and demographic data including age, BMI, gender, vaccination status, vaccination type, days since last vaccine dose, race, WHO initial disease severity, diabetes status, and days since symptom onset were collected. We used EMR review to assess the presence of 12 symptoms: fatigue, shortness of breath, palpitations, chest pain, brain fog, joint pain, myalgia, headaches, gastrointenstinal (GI) symptoms, dizziness, cough, and anosmia. We selected these symptoms as they have been used to define long COVID symptom clusters previously (18, 20).

### 2.2 Measurement of biomarkers

Plasma levels of 42 biomarkers were measured which included systemic inflammatory markers IFN-γ, IL-2, IL-6, IL-12p70, TNF-α, CRP, IFN-β and IL-17A, innate inflammatory markers IL-1α, IL-28B/IFN-λ3, sCD14, CD163, LBP, MMP-2, MMP-9, PTX3, and TIMP-1, chemokines IL-8, IP-10, I-TAC, CCL-1, MCP-1, MIP-1α, MIP-1β, and MIP-3b, immune cell growth factors G-CSF, GM-CSF, FGF, TPO, and VEGF, endothelial activation markers sICAM-1, vWF, and E-Selectin, anti-inflammatory cytokines IL-4, IL-10, and IL-13, microbial translocation markers Zonulin and β-D-glucan, coagulation markers GPVI and D Dimer, heart failure marker NT-proBNP and neuronal injury marker S100B. All biomarker levels were measured using kits from Meso Scale Discovery (Rockville, Maryland), except Zonulin, β-D-glucan, GPVI, and D Dimer, which were measured using ELISA kits from ALPCO (Salem, NH), Associates of Cape Cod (East Falmouth, MA), Thermo Fisher Scientific (Waltham, MA) and Diagnostica Stago (Asnières, France), respectively. All panels were performed according to the manufacturer’s instructions.

### 2.3 Measurement of binding antibody

Binding IgG against Spike and Nucleocapsid proteins of SARS-COV-2 were measured using the V-PLEX SARS-CoV-2 Panel 25 IgG Kit (Catalog No. K15583U-2) and V-PLEX COVID-19 Coronavirus Panel 2 IgG Kit (Catalog No. K15369U-2 from Meso Scale Discovery (Rockville, Maryland). Assays were performed according to manufacturer instructions. Results were analyzed using MSD Discovery Workbench 4.0.12. and reported in arbitrary units (AU)/mL. Levels of 1000 AU/mL for anti-spike and 5000 AU/mL for anti-nucleocapsid were used as cut-offs for positivity based on historical controls collected at our institution prior to the pandemic.

### 2.4 Statistical analysis

We used principal component analysis (PCA) to reduce dimensionality of the 42 biomarker levels. PCs representing 80% of variance were then hierarchically clustered using Ward’s method with a Euclidian distance measure to construct biomarker derived clusters. To derive symptom clusters, we adopted methodology which has been described in detail elsewhere (18, 20). Briefly we used multiple correspondence analysis (MCA) to remove dimensionality of the dataset, and hierarchical clustering on the results of the MCA using squared Euclidean distance and Ward’s minimum variance linkage.

We used univariable and multivariable unadjusted multinomial logistic regression models to explore associations of biomarker clusters, considering variables that have been associated with risk of long COVID including age, sex, ethnicity, BMI, vaccination status, days since symptom onset, initial disease severity, and diabetes. Categorical variables including symptom prevalence, gender, initial disease severity, vaccination status, ethnicity, and diabetes status were analyzed using the chi-square test. Continuous variables including age, BMI, days since last vaccination dose, and days since symptom onset were analyzed using the Kruskal-Wallis test. Age, sex, and ethnicity were included in multivariate models a priori, in addition to demographics variables that were found to be significantly different amongst clusters. All analysis was carried out using R version 4.1.2 and GraphPad Prism version 9.5.0.

## 3 Results

### 3.1 Study cohort

A total of 101 individuals were recruited for this study from January 2021 to August 2021. 97 (96%) of individuals were confirmed positive for COVID-19 infection by a PCR test, point-of-care rapid test, or antigen test, and the remainder were confirmed to have been previously infected by the presence of a positive nucleocapsid antibody on serologic testing. Median duration of symptoms was 160 (IQR: 104-251.50) days preceding sample collection None of the individuals had been vaccinated at the time of infection. 62 (61.39%) of individuals received at least one dose of vaccination before sample collection. 31 (30.69%) of the individuals were male and the cohort had a median age of 47 (IQR 36-55) years.

### 3.2 Plasma biomarkers reveal three distinct inflammatory clusters

**Figure 1:**
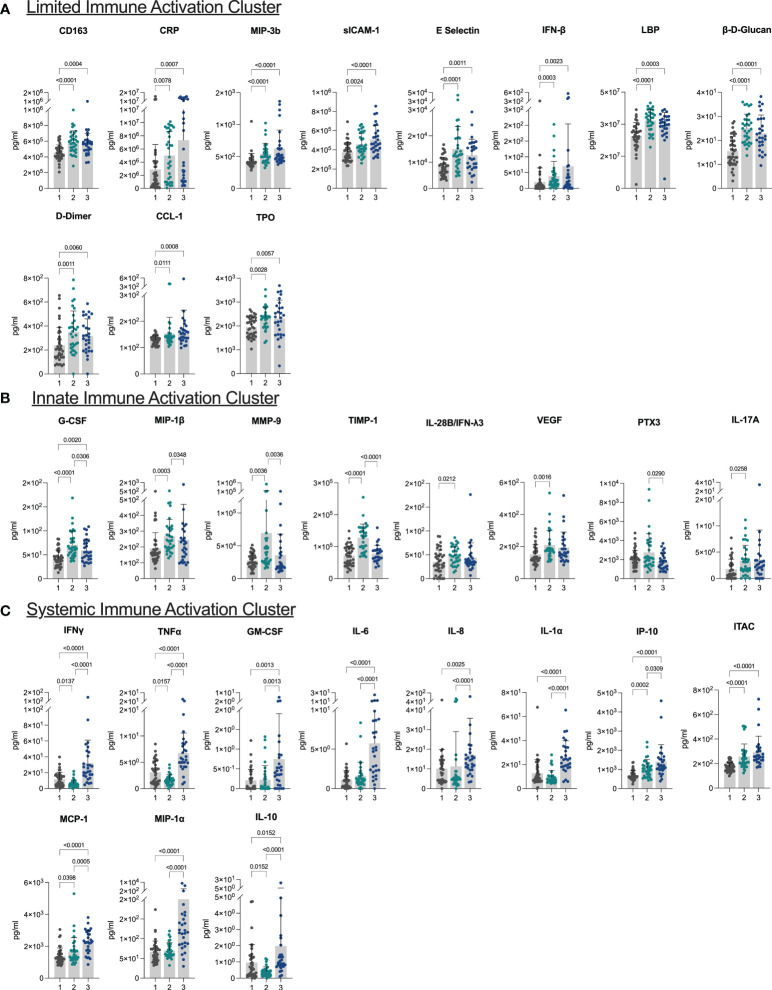
Plasma biomarkers reveal three distinct inflammatory clusters **(A–C)** Bar plots demonstrating differences in levels of biomarkers characteristic of each of the three clusters. Cluster 1: limited immune activation cluster, cluster 2: innate immune activation cluster, and cluster 3: systemic immune activation cluster.

Principal component analysis and hierarchical clustering revealed three distinct biomarker clusters (**Supplementary Figure 1**). The first cluster was the largest of the three clusters with 40 (39.60%) individuals. Overall, this cluster was characterized by low levels of inflammation and innate immune activation, and thus was termed the limited immune activation cluster. Compared to the other two clusters, this cluster had lower levels of immune markers including CD163, CRP, sICAM-1, E Selectin, IFNβ, LBP, β-D-Glucan, D-Dimer, and TPO, and chemokines MIP-3b and CCL-1 (all p <0.05, **Figure 1A**). This cluster was the youngest, with a median age of 39.00 (IQR: 29.25 – 52.00) years and had the lowest BMI (median 25.55 (IQR: 21.77 – 30.38)) of the three clusters (**Table 1**).

**Table 1:** Demographic differences between clusters.

	Total (n = 101)	Limited Immune Activation (n = 40)	Innate Immune Activation (n = 33)	Systemic Immune Activation (n = 28)	p-value
**Age (years) (median (IQR))**	47 (36 – 55)	39.00 (29.25 – 52.00)	47.00 (39.50 – 56.00)	51.50 (41.75 – 58.00)	**0.010**
**Sex Male Female**	30.69% (31) 69.31% (70)	32.50% (13) 57.40% (27)	36.36% (12) 63.64% (21)	21.43% (6) 78.57% (22)	0.430
**Ethnicity White African American Other**	59.41% (60) 19.80% (20) 20.79% (21)	70.00% (28) 12.50% (5) 17.50% (7)	51.52% (17) 24.24% (8) 24.24% (8)	53.57% (15) 25.00% (7) 21.43% (6)	0.485
**BMI (kg/m^2^) (median (IQR))**	29.24 (24.73 – 34.70)	25.55 (21.77 – 30.38)	32.24 (28.34 - 37.24)	31.21 (25.11 – 35.96)	**<0.001**
**Diabetes**	13.86% (14)	12.50% (5)	9.09% (3)	21.43% (6)	0.362
**WHO Acute Disease Severity Mild Moderate Severe Critical**	77.23% (78) 12.87% (13) 5.94% (6) 3.96% (4)	82.50% (33) 10.00% (4) 2.50% (1) 5.00% (2)	72.73% (24) 15.15% (5) 9.09% (3) 3.03% (1)	75.00% (21) 14.29% (4) 7.14% (2) 3.57% (1)	0.891
**Days Since Symptom Onset (median (IQR))**	160.00 (IQR: 104.00 – 251.50)	186.50 (IQR: 125.75 – 309.25)	127.00 (IQR: 86.00 – 171.50)	202.00 (IQR: 128.00 – 290.00)	**0.006**
**Vaccinated (post COVID infection)**	61.39% (62)	70.00% (28)	24.24% (8)	92.86% (26)	**<0.001**
**Vaccine Type Pfizer Moderna J&J**	31.68% (32) 25.74% (26) 3.96% (4)	32.50% (13) 35.00% (14) 2.50% (1)	12.12% (4) 12.12% (4) 0.00% (0)	53.57% (15) 28.57% (8) 10.71% (3)	0.465
**Days Since Last Vaccine Dose (median (IQR))**	62 (29.00-93.50)	67 (27.75-94.75)	62 (44.50-93.25)	57 (32.25-82.75)	0.869

BMI, body mass index; WHO, World Health Organization.Significant (p <0.05) values are bolded.

The next cluster consisted of 33 (32.67%) individuals and presented with higher levels of markers of innate immune activation compared to the low inflammatory response cluster. Levels of G-CSF, MIP-1β, MMP-9, and TIMP-1 were elevated in this cluster compared to both other clusters, and IL-28B/IFNλ3, VEGF, and IL-17A were elevated in this cluster compared to the limited immune activation cluster (all p <0.05) (**Figure 1B**). This cluster was termed the innate immune activation cluster as G-CSF and MIP-1β are key innate immune cytokines, and MMP-9 leads to release of innate immune cytokines in its role in the degradation of extracellular matrix (21). This cluster was older (median age 47.00 (IQR: 39.50 – 56.00) years), had a higher BMI [median 32.24 (IQR: 28.34 - 37.24)] than the limited immune activation cluster. It also had the lowest number of individuals who had been vaccinated prior to sample collection (24.24%) and had the shortest duration of symptoms at the time of sample collection (median 127.00 (IQR: 86.00 – 171.50) days from symptom onset) (**Table 1**).

The last cluster, which we termed a systemic immune activation cluster, comprised the remaining 28 (27.72%) individuals and showed high levels of most markers including systemic inflammation markers IFNγ, TNFα, IL-6, pro-inflammatory chemokines IL-8, IP-10, ITAC, MCP-1, and MIP-1α, growth factor GM-CSF, and the innate inflammatory marker IL-1α. The anti-inflammatory marker IL-10 was also upregulated (**Figure 1C**). However, PTX3, another innate immune cytokine was lower than in the innate immune cluster. This cluster was the oldest (median age 51.50 (IQR: 41.75 – 58.00) years), although age was not significantly different than the innate immune activation cluster (p >0.05). Similarly, BMI (median 31.21 (IQR: 25.11 – 35.96)) was significantly higher than the limited immune activation cluster, but not significantly different to the innate immune activation cluster. This cluster had the highest proportion of individuals that had been vaccinated prior to sample collection (92.86%) and was sampled longest post symptom onset of the three clusters (median 202.00 (IQR: 128.00 – 290.00) days) (**Table 1**).

### 3.3 Inflammatory profile does not correlate with long COVID symptoms

We next investigated the correlation between the prevalence of symptoms and inflammatory clusters. Shortness of breath and fatigue were the most prevalent symptoms among the cohort, occurring in 72.23% and 48.51% respectively. GI symptoms and joint pain, which were present in 7.92% and 8.91% were the least prevalent. MCA and hierarchical clustering revealed 3 symptoms clusters, similar to those demonstrated elsewhere (**Supplementary Figure 2**, **Supplementary Table 1**) (18, 20). The first cluster [n=17 (16.83%)], a musculoskeletal (MSK) or pain symptom cluster, was characterized by high levels of headache (64.71%), brain fog (82.35%), joint pain (41.18%), myalgia (47.06%) and fatigue (82.35%) compared to the other two clusters, and had the highest number of symptoms with a median of 6 (IQR: 5-6) symptoms per participant. The second cluster [n=47 (46.53%)], a cardiorespiratory cluster, had high proportions of shortness of breath (100%), chest pain (59.57%) and palpitations (42.55%) compared to the other two clusters, and had a median of 3 (IQR: 2-4.5) symptoms per participant. The last cluster, a less symptomatic cluster [n=37 (36.63%)], had a median of 1 (IQR: 1-2) symptoms per participant, and cough (29.72%), was the only symptom more prevalent in this cluster compared to the other two clusters.

However, looking at the correlation with inflammatory clusters, we found no significant difference in the prevalence of symptom cluster between inflammatory cluster, nor significant differences in the prevalence of any of the 12 symptoms between inflammatory clusters (all p >0.05 **Table 2**).

**Table 2:** Prevalence of symptoms.

	Total (n = 101)	Cluster 1 (n = 40)	Cluster 2 (n = 33)	Cluster 3 (n = 28)	p-value
**MSK/pain**	16.83% (17)	10% (4)	18.18% (6)	25% (7)	0.26
**Cardiorespiratory**	46.53% (47)	52.5% (21)	51.52% (17)	32.14% (9)	0.2
**Less symptomatic**	36.63% (37)	37.5% (15)	30.3% (10)	42.86% (12)	0.59
**Fatigue**	48.51% (49)	35.00% (14)	60.61% (20)	53.57% (15)	0.076
**Shortness of Breath**	72.23% (73)	77.50% (31)	69.70% (23)	67.86% (19)	0.629
**Palpitations**	26.73% (27)	35.00% (14)	24.24% (8)	17.86% (5)	0.269
**Chest Pain**	36.63% (37)	37.50% (15)	39.39% (13)	32.14% (9)	0.833
**Brain Fog**	33.66% (34)	30.00% (12)	33.33% (11)	39.29% (11)	0.727
**Joint Pain**	8.91% (9)	7.50% (3)	6.06% (2)	14.29% (4)	0.490
**Myalgia**	18.81% (19)	10.00% (4)	30.30% (10)	17.86% (5)	0.086
**Headache**	15.84% (16)	10.00% (4)	21.21% (7)	17.86% (5)	0.402
**GI Symptoms**	7.92% (8)	7.50% (3)	6.06% (2)	10.71% (3)	0.792
**Dizziness**	13.86% (14)	22.5% (9)	6.06% (2)	10.71% (3)	0.110
**Cough**	15.84% (16)	15.00% (6)	12.12% (4)	21.43% (6)	0.601
**Anosmia**	9.90% (10)	7.50% (3)	15.15% (5)	7.14% (2)	0.468

MSK, musculoskeletal; GI, gastrointestinal.

### 3.4 Association between clinical variables and inflammatory clusters

To further investigate the association between clinical variables and cluster membership, we constructed multinomial logistic regression models considering variables that may affect host inflammatory response (**Table 3**).

**Table 3:** Logistic regression model results.

	Univariate			Multivariate		
	Odds Ratio	Conf. Interval.	P-value	Odds Ratio	Conf. Interval.	P-value
Age						
Limited	ref	–	–	ref	–	–
Innate	**1.05**	**1.01 – 1.09**	**0.027**	1.05	1.05	0.087
Systemic	**1.07**	**1.02 – 1.11**	**0.005**	**1.06**	**1.02 – 1.12**	**0.011**
Male Sex						
Limited	ref	–	–	ref	–	–
Innate	1.19	0.45 – 3.13	0.729	1.91	0.53 – 6.86	0.324
Systemic	0.57	0.19 – 1.74	0.320	0.55	0.16 – 1.87	0.340
Non-Caucasian Ethnicity						
Limited	ref	–	–	ref	–	–
Innate	2.20	0.84 – 5.74	0.109	1.59	0.40 – 6.28	0.506
Systemic	2.02	0.74 – 5.52	0.169	1.23	0.35 – 4.26	0.746
BMI						
Limited	ref	–	–	ref	–	–
Innate	**1.11**	**1.04 – 1.20**	**0.004**	**1.15**	**1.04 – 1.27**	**0.006**
Systemic	**1.08**	**1.01 – 1.17**	**0.029**	1.06	0.97 – 1.15	0.211
Vaccinated						
Limited	ref	–	–	ref	–	–
Innate	**0.14**	**0.05 – 0.39**	**<0.001**	**0.09**	**0.02 – 0.38**	**0.001**
Systemic	**5.57**	**1.14 – 27.30 **	**0.034**	**5.95**	**1.02 – 34.75 **	**0.048**
Days Since Symptom Onset						
Limited	ref	–	–	ref	–	–
Innate	**0.99**	**0.99 – 1.00**	**0.015**	1.00	0.99 – 1.00	0.295
Systemic	1.00	1.00 – 1.01	0.657	1.00	0.99 – 1.00	0.433

Significant (p <0.05) values are bolded.

In univariate analysis, older age and higher BMI were associated with an increased odds of being in the innate immune activation cluster (OR for age: 1.05, 95% CI: 1.01-1.09, p= 0.027, OR for BMI: 1.11, 95% CI: 1.04-1.20, p= 0.004), while vaccination status and increasing time from symptom onset were associated with reduced odds of being in the innate immune activation cluster when compared to the limited immune activation cluster (vaccination OR: 0.14, 95% CI: 0.05-0.39, p<0.001, time from symptom onset OR 0.99, 95%CI: 0.99-1.00, p= 0.015). After multivariate adjustment, vaccination remained significantly associated with a reduced odds of being in the innate immune activation cluster (OR: 0.09, 95% CI: 0.02-0.38, p= 0.001) while BMI remained associated with higher odds of belonging to this cluster (OR: 1.15, 95%CI: 1.04-1.27, p= 0.006).

Looking next at the systemic immune response cluster, in univariate analysis, older age (OR: 1.07, 95% CI: 1.02-1.11, p= 0.005), higher BMI (OR: 1.08, 95% CI: 1.01-1.17, p= 0.029), and vaccination status (OR: 5.57, 95% CI: 1.14-27.30, p= 0.034) were significantly associated with increased odds of being in the systemic immune response cluster when compared to the limited immune response cluster. After multivariate adjustment, the associations with vaccination (OR: 5.95, 95%CI: 1.02-34.75, p= 0.048) and age remained (OR: 1.06, 95%CI: 1.02-1.12, p= 0.011), while that with BMI did not.

### 3.5 Differences in serologic response between clusters

**Figure 2:**
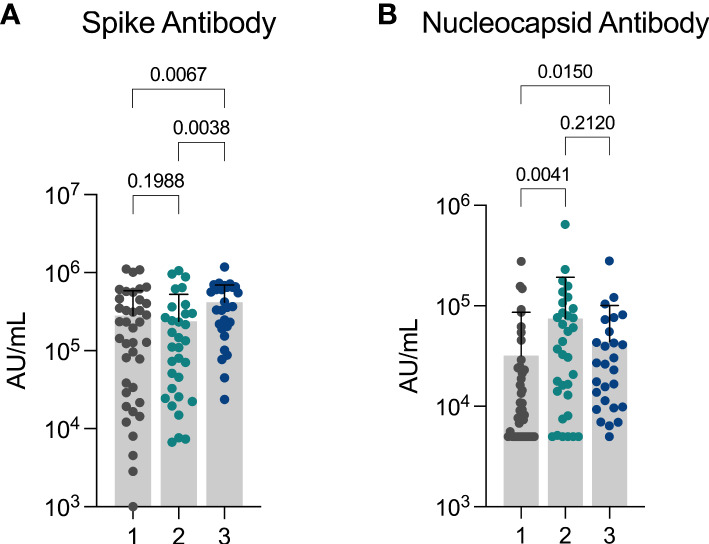
Differences in anti-spike and anti-nucleocapsid response between clusters **(A)** Anti-Spike and **(B)** Anti-Nucleocapsid levels observed in cohort showing differences observed between three inflammatory clusters. Cluster 1: limited immune activation cluster, cluster 2: innate immune activation cluster, and cluster 3: systemic immune activation cluster.

We next measured anti-spike and anti-nucleocapsid levels in available samples (n=99). Anti-spike antibodies were detectable in 98 (98.99%) individuals: 38 out of 39 (97.44%) in the limited immune activation cluster (median 195,000 AU (IQR: 28,900 AU – 448,000 AU), 32 out of 32 (100.00%) in the innate immune activation cluster (median 123,000 AU (IQR: 27,400 AU – 298,000 AU), and 28 out of 28 (100.00%) in the systemic immune activation cluster (median 364,000 AU, IQR: 19,400 AU – 650,000 AU). Anti-spike antibody levels were significantly greater in the systemic immune activation cluster when compared to both other clusters (**Figure 2A**), as expected given the higher vaccination rate.

Anti-nucleocapsid antibodies were detected in 81 (81.82%) individuals (n = 99): 27 out of 39 (69.23%) in the limited immune activation cluster (median 9,260 AU (IQR: 5,000 AU – 24,800 AU), 28 out of 33 (84.85%) in the innate immune activation cluster (median: 37,100 AU (IQR: 10,500 AU – 89,700 AU), and 26 out of 27 (96.30%) in the systemic immune activation cluster (median: 26,300 AU (IQR: 9,860 AU – 55,500 AU). Anti-nucleocapsid antibody levels were significantly greater in both the innate inflammatory cluster and the systemic inflammatory cluster in comparison to the low inflammatory response cluster (**Figure 2B**).

## 4 Discussion

In this study, we identify three distinct inflammatory profiles in individuals with long COVID. These profiles featured contrasting levels of inflammation and had unique clinical and demographic associations. The first cluster featured low levels of inflammation, was the youngest and had the lowest BMI. The second cluster, an “innate immune activation” cluster was independently associated with higher BMI and the last cluster, a “systemic inflammation” cluster was independently associated with older age. However, we did not observe differences in symptom profile across inflammatory clusters, emphasizing the need to control for clinical variables when investigating the pathophysiology of long COVID.

Previous studies have proposed systemic inflammation as a cause of long COVID, and have implicated cytokines that were measured in this study. Elevation in serum levels of IL-1β, IL-6, and TNFα have been suggested as a hallmark of long COVID (15), but this study did not present the demographic differences between those with and without long COVID. Another study which included age and BMI matched SARS-CoV-2 convalescent controls, demonstrated higher IP-10 and TNFα in early recovery (a median of 52 days post symptom onset) and higher IL-6 in late recovery (a median of 124 days post symptom onset) in long COVID (17). At the early time point, IL-6, IFNγ, IL-10 and TNFα were higher in those with the greatest number of symptoms. Despite higher levels of these cytokines in the systemic immune activation cluster, symptoms were not more frequent in this analysis. More recently, a study demonstrated an inflammatory subcluster of long COVID, with upregulated IL-8, IL-6 and IL-1β, similar to the systemic inflammation cluster observed in our study, but once again this cluster was older and a clear correlation with increased symptoms was not demonstrated (22). Overall, the role of inflammatory cytokines in long COVID symptoms remains uncertain.

We observed higher levels of nucleocapsid antibodies in the innate immune activation and systemic inflammation cluster compared to the limited immune activation cluster. Nucleocapsid antibodies correlate with initial disease severity but have also been used as a proxy measurement for the existence of a persistent reservoir of viral antigen, which could drive some of the inflammation seen in these two more inflamed clusters (23, 24). Interestingly, these clusters had different rates of vaccination, even when controlling for potential confounders. Studies have shown that vaccination against SARS-CoV-2 may reduce symptoms of long COVID (6, 7), with clearance of a viral reservoir proposed as a mechanism for this improvement (25). Here, the highest vaccination level was seen in the most inflamed group. Whether this could represent an could reflect an inflammatory response that would lead to clearance of a viral reservoir requires further study. Reassuringly, there was no increase in symptoms seen in this more vaccinated group.

Unsupervised clustering of symptoms demonstrated a pain-predominant cluster, a cardiorespiratory cluster, and a less symptomatic cluster. Previous studies in a long COVID cohort based in Ireland have found similar symptom phenotypes and demonstrated greater functional impact associated with the pain and cardiorespiratory phenotypes (18). While the replication of these phenotypes across independent populations supports the potential for their clinical relevance, we did not identify differences in inflammatory profile. A number of other causes have been hypothesized to cause long COVID, including changes in levels of B cells and T cells, presence of autoantibodies, reactivated viruses, and inadequate antibody production (1). Further research is needed to determine whether these factors are associated with clinical phenotype.

Our study, while providing valuable insights, had certain limitations that warrant consideration. Firstly, we acknowledge that our analysis was constrained by the small sample size, particularly in the systemic immune activation cluster where only two unvaccinated individuals were included. This limited representation may have limited our ability to model the effect of vaccination on cluster membership. Moreover, symptoms that have been previously observed to be commonly associated with long COVID showed a relatively low prevalence in this cohort, with fatigue being present in only 48.51% of individuals and brain fog in 33.66%. This may indicate that individuals in the cohort had a mild presentation of long COVID compared to other published cohorts, and may have limited the ability to detect differences in symptoms between inflammatory clusters. Finally, this study is cross sectional, preventing analysis of inflammatory phenotype on trajectory of long COVID symptoms.

In summary, this study demonstrates inflammatory profiles in a long COVID cohort and the association with clinical variables. Further research is needed to determine the pathophysiologic changes that underlie different long COVID presentations.

## Data availability statement

The raw data supporting the conclusions of this article will be made available by the authors, without undue reservation.

## Ethics statement

The studies involving humans were approved by Rush University Institutional Review Board Office of Research Affairs. The studies were conducted in accordance with the local legislation and institutional requirements. The participants provided their written informed consent to participate in this study.

## Author contributions

SD: Conceptualization, Formal analysis, Writing – original draft, Writing – review & editing, Methodology, Investigation. JF: Conceptualization, Writing – original draft, Methodology, Investigation, Data curation. GC: Conceptualization, Writing – review & editing. PM: Conceptualization, Methodology, Supervision, Writing – review & editing. HW: Conceptualization, Writing – review & editing. JM: Conceptualization, Methodology, Supervision, Writing – review & editing. AL: Supervision, Methodology, Investigation, Data curation, Conceptualization, Writing – review & editing, Writing – original draft. GK: Writing – original draft, Writing – review & editing, Conceptualization, Data curation, Formal analysis, Investigation, Methodology, Supervision.
