# Supplementary material for: Identification of inflammatory clusters in long-COVID through analysis of plasma biomarker levels
Source: Front Immunol. 2024 Apr 30;15:1385858. doi: 10.3389/fimmu.2024.1385858 (PMC11091280; doi:10.3389/fimmu.2024.1385858)
Supplement: Supplementary file 1 [file DataSheet_1.docx]

**Supplementary table 1 – Prevalence of symptoms between clusters**

|  | **MSK/Pain** | **Cardiorespiratory** | **Less Symptomatic** |
| --- | --- | --- | --- |
| **Fatigue** | 14 (82.35%) | 29 (61.7%) | 6 (16.22%) |
| **Shortness of Breath** | 12 (70.59%) | 47 (100%) | 14 (37.84%) |
| **Palpitations** | 3 (17.65%) | 20 (42.55%) | 4 (10.81%) |
| **Chest pain** | 7 (41.18%) | 28 (59.57%) | 2 (5.41%) |
| **Brain fog** | 14 (82.35%) | 7 (14.89%) | 13 (35.14%) |
| **Joint pain** | 7 (41.18%) | 2 (4.26%) | 0 (0%) |
| **Myalgia** | 8 (47.06%) | 11 (23.4%) | 0 (0%) |
| **Headache** | 11 (64.71%) | 4 (8.51%) | 1 (2.7%) |
| **GI symptoms** | 5 (29.41%) | 2 (4.26%) | 1 (2.7%) |
| **Dizziness** | 7 (41.18%) | 6 (12.76%) | 1 (2.7%) |
| **Cough** | 2 (11.76%) | 3 (6.38%) | 11 (29.72%) |
| **Anosmia** | 7 (41.18%) | 0 (0%) | 3 (8.11%) |

**Supplemental Figure 1:** Heatmap depicting inflammatory clusters derived from hierarchical clustering of 42 plasma biomarkers in individuals with long COVID. Red represents high plasma levels, while blue represents low plasma levels. Cluster 1: limited immune activation cluster, cluster 2: innate immune activation cluster, and cluster 3: systemic immune activation cluster.


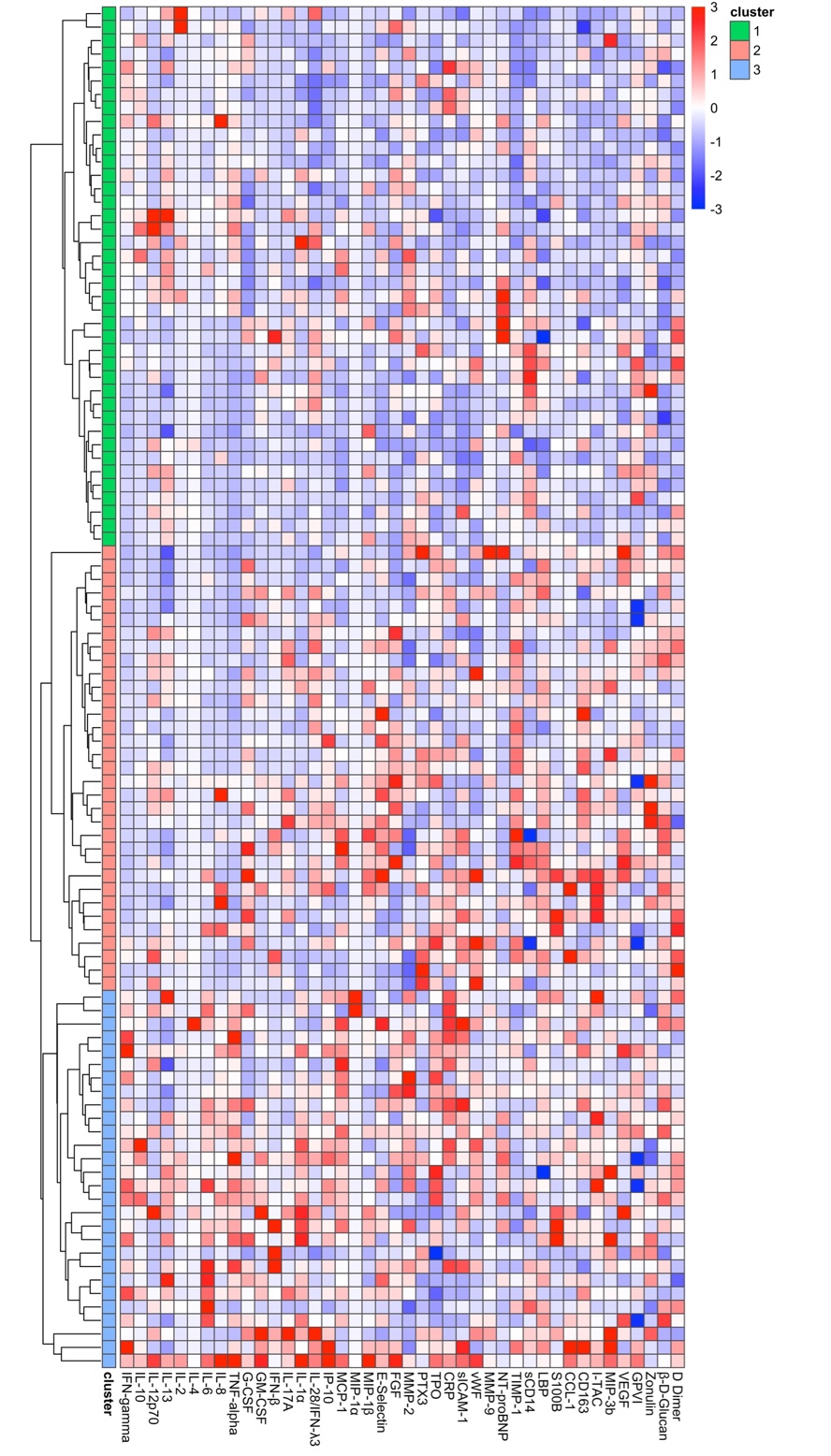


**Supplementary Figure 2** – Heatmap demonstrating symptom clusters derived from prevalence 12 symptoms among cohort.

Legend: Red represents presence of a symptom. SOB – shortness of breath; GI – gastrointestinal; MSK – musculoskeletal.
